# On Progressive Locomotor Ataxy

**Published:** 1866-07

**Authors:** Benjamin Durham

**Affiliations:** Chicago, Ill.


### CHICAGO MEDICAL JOURNAL.

VOL. XXIII. JULY, 1866. No. 7

### ORIGINAL CONTRIBUTIONS.

*On Progressive Locomotor Ataxy.* By BENJAMIN DURHAM, Jr., M. D., Chicago, Ill.

The study of diseases of the nervous system is receiving much attention, especially since the investigations of Brown-Sequard, and the lines of discrimination in diagnosis are drawn more strictly than heretofore. By this means those most skilled in this specialty have separated the cases of progressive locomotor ataxy from the old class of tabes dorsalis; and though physicians in general practice may rarely meet with instances of this disease, yet its study may help us to diagnose more accurately the usual cases of spinal paralysis. [Authors consulted: Aitken, Brown-Sequard and Flint; Duchenne, in *Year Books of New Sydenham Soc.;* Stille and Topinard, in *Am. Jour. of Med. Sci.;* Althaus, Radcliffe and Ramskill, in *London Lancet.*]

In 1858, M. Duchenne (de Boulogne) described a form of paralysis where the patient retained the appearance of health and muscular development without being able to perform combined voluntary movements. In his case (a painter, aged 28) there was double but incomplete paralysis of the sixth pair of nerves, characteristic boring and flying pains, integrity of the muscular force, but complete loss of co-ordination of the lower limbs, with diminished sensibility. This patient died of another disease, but at the autopsy no appreciable lesion in the brain or spinal cord was detected. During the following year Duchenne published his observations, and described "ataxie locomotrice progressive as a disease whose chief characters are the increasing loss of the co-ordinating power of the movements, and retention of muscular power in strong contrast to the apparent paralysis." He believed the function of co-ordination is primarily affected, and located the seat of the disorder in the cerebellum, corpora quadrigemina and the intervening commissure. Since the time of these observations the subject has been fully investigated by English and German physicians, who find that the present disease has formerly been included under the head of spinal paralysis and generally confounded with tabes dorsalis.

The pathological changes in the nervous centres were only determined by the use of the microscope. The anatomical lesions occur first in the periphery of the cerebral nerves, then in the posterior columns of the spinal cord and the posterior roots of the spinal nerves, and sometimes in the optic nerves, motores oculi and tubercula quadrigemina. With these appearances may be associated congestion or chronic inflammation of the spinal cord and its meninges, and degeneration of the central gray matter, but primarily this disease consists in a wasting away of the medullary matter in the nervous tubules and granulation with hypertrophy of the intermediate elements. In place of the nerve fibres there are delicate empty tubes surrounded by cells containing several nuclei. In all cases there exists a gutter-like depression in the posterior portion of the cord, quite marked in the dorsal and lumbar regions. Bourdon gives the anatomical characters as follows: "The posterior columns of the spinal cord, and sometimes the cineritious substance also, had undergone degeneration, were of a grayish-yellow color and semi-transparent; the posterior roots of the spinal nerves were also atrophied, sometimes the optic nerve, and in one case the tubercula quadrigemina had become altered. Anatomically, the disease is a chronic inflammatory process, resulting in the atrophy of the nerve elements, and which, being confined to the posterior column of the spinal cord, begins in its lumbar region, and gradually extends thence both upwards and downwards. With this alteration is associated a spinal meningitis affecting the posterior face of the cord, and proportioned in its degree to that of the alteration of the cord itself." These pathological appearances agree with the symptoms of the disease and the discovery of Brown-Sequard that "the posterior columns are the principal channels for the excitations which produce reflex movements."

"In cases of alterations limited to the posterior columns, but occupying all their length and thickness, or only the whole of the lumbar swelling, there is an impossibility of standing or walking, depending upon the loss of the reflex actions of the limbs; but, in bed, the patients in such cases can move their lower limbs pretty freely." In limited affections of these columns there is hyperæsthesia in the parts of the body behind the injury, and the reflex actions and voluntary movements of the limbs may persist, but in alterations of a great part of their length the hyperæsthesia is much less. So in the earlier stages of progressive locomotor ataxy we have fugitive pains, which are referred to a rheumatic or neuralgic origin, and slight affection of some of the cerebral nerves as the sixth or third pair, and in certain cases impairment of vision. After some time the patient notices a difficulty in walking, his motions become uncertain, often he cannot walk without watching the position of his feet, and he acquires a peculiar, awkward, staggering gait, so that his legs are thrown widely apart, while the body is swayed from side to side, and though when lying down he possesses full muscular power, yet his uncertain movements make walking very tiresome.

Trousseau relates one instance where a physician requested him to see an odd case of paralysis in a man eighty years old. Though unable to walk, the patient overthrew his unsuspecting attendant, who tried to hold his flexed leg in such a way that he could not extend it. The power remains without the control of muscular action. The character of the disease appears when any attempt is made to execute a combined movement, especially when the actions are not guided by the sight. In the progress of the affection the upper extremities become affected, which is soonest manifested by inability to control the fingers in buttoning the clothing, particularly at the neck or back where vision fails to assist in directing the movements. Later in the disease there is diminution of tactile sensibility, the power of speech becomes impaired, the articulation is indistinct and stammering, and sometimes the use of the eyes is lost. In the female the catamenia and the power of conception continue, but in the male there seems at first to be an increase of sexual desire, which, with the virile power, is lost in the progress of the degeneration ; sometimes there is incontinence of urine, but generally the urinary and digestive organs present no special symptoms. In the later stages the disease may be complicated with symptoms of motor paralysis and chronic myelitis, which sometimes coexist with this affection, and the patient may lapse into paralysis or insanity.

It was formerly supposed that ataxy was caused by onanism or excessive sexual indulgence, but there is no special origin for this nervous disorder. The primary cause of progressive locomotor ataxy operates upon the whole nervous system, with a preference for the spinal cord, and consists in an alteration of the reflex power, which is sooner manifested in the lower than the upper limbs, since this reflex co-ordinating power is more developed in the lumbar portion of the cord. There may be a hereditary tendency to this disease, but sex exerts no influence; exposure to cold and dampness, bodily and mental fatigue, and a rheumatic condition, have been mentioned as causes.

In regard to the diagnosis, the physician is liable to confound this special form with chronic myelitis and general paralysis. Many cases have been considered as instances of incurable chorea, which they resemble in many of their symptoms. But though the type of the spinal disease may not be distinguished by the premonitory signs, yet it will appear plainly when in the progress of the case there is a defect of co-ordinating power without wasting or loss of action of the muscles.

In regard to the prognosis, it is always very unfavorable; still, the affection is a lingering one, and the patient usually sinks from some intercurrent disease: a hope may be held out that with proper care the progress of the degeneration may be stayed, but great caution is necessary in regard to promising any return to health.

In respect to the treatment, the different remedies used in other nervous disorders have been employed without eliciting much confidence. The indications of treatment are, 1st, to combat any complication which aggravates the disease; 2dly, to enrich the blood, and thus relieve the constitutional affection; and 3dly, to assist the nutrition of the spinal cord. Hygienic remedies must be the physician's principal weapons. Attention has been called to the judicious employment of electricity and the use of sulphur baths, which in some cases have been productive of a little improvement, but hydropathy has been faithfully used without any benefit. The different remedies which have gained a transient reputation in the treatment of ataxy seem to have been used in cases attended with myelitis, meningitis, or a rheumatic diathesis. We can readily understand the benefit which in such instances might attend the use of belladonna, ergot and bromide of potassium in relieving the congestion of the spinal cord, and though the action of nitrate of silver is not so clearly defined, yet it seems to act much in the same way, and certainly proves useful in cases of a rheumatic character, where, too, iodide of potassium has been beneficial. But though the pathological changes are local, we must not forget that these are the manifestations of constitutional perversions of nutrition, and every means is necessary to enrich the blood; therefore, the patient should live on the most nutritious food and be much in the open air, while medicinally we may resort to the hypophosphite of soda and cod liver oil. Lastly, in attempting to assist the nutrition of the spinal cord, we can increase the flow of blood to this organ by a very guarded use of strychnine or opium, using the precaution not to produce an injurious congestion of the cord. It seems as yet to be an open question whether nitrate of silver and iodide of potassium have any power to check the degenerative changes which impair the nutrition of this nervous centre. In cases unmixed with inflammation some good may be experienced by position, causing the patient when in bed to lie on his back with the lower extremities raised higher than the spine, and in suitable cases we might resort to the alternation of heat and cold, by placing ice on the back for ten minutes and following with a hot poultice for an hour. The fatal termination of the cases noticed has usually been from some disorder of the respiratory system or from typhoid fever, and death rarely occurs without the supervention of some other disease.

